# A Spontaneous Animal Model of Intestinal Dysmotility Evoked by Inflammatory Nitrergic Dysfunction

**DOI:** 10.1371/journal.pone.0095879

**Published:** 2014-05-12

**Authors:** Tatsuhiro Masaoka, Tim Vanuytsel, Christophe Vanormelingen, Sebastien Kindt, Shadea Salim Rasoel, Werend Boesmans, Gert De Hertogh, Ricard Farré, Pieter Vanden Berghe, Jan Tack

**Affiliations:** 1 Translational Research Center for Gastrointestinal Disorders, University of Leuven, Leuven, Belgium; 2 Department of Pathology, University of Leuven, Leuven, Belgium; University of California, Los Angeles, United States of America

## Abstract

**Background and Aims:**

Recent reports indicate the presence of low grade inflammation in functional gastrointestinal disorders (FGID), in these cases often called “post-inflammatory” FGIDs. However, suitable animal models to study these disorders are not available. The Biobreeding (BB) rat consists of a diabetes-resistant (BBDR) and a diabetes-prone (BBDP) strain. In the diabetes-prone strain, 40–60% of the animals develop diabetes and concomitant nitrergic dysfunction. Our aim was to investigate the occurrence of intestinal inflammation, nitrergic dysfunction and intestinal dysmotility in non-diabetic animals.

**Methods:**

Jejunal inflammation (MPO assay, Hematoxylin&Eosin staining and inducible nitric oxide synthase (iNOS) mRNA expression), *in vitro* jejunal motility (video analysis) and myenteric neuronal numbers (immunohistochemistry) were assessed in control, normoglycaemic BBDP and diabetic BBDP rats. To study the impact of iNOS inhibition on these parameters, normoglycaemic BBDP rats were treated with aminoguanidine.

**Results:**

Compared to control, significant polymorphonuclear (PMN) cell infiltration, enhanced MPO activity, increased iNOS mRNA expression and a decreased ratio of nNOS to Hu-C/D positive neurons were observed in both normoglycaemic and diabetic BBDP rats. Aminoguanidine treatment decreased PMN infiltration, iNOS mRNA expression and MPO activity. Moreover, it restored the ratio of nNOS to Hu-C/D positive nerves in the myenteric plexus and decreased the abnormal jejunal elongation and dilation observed in normoglycaemic BBDP rats.

**Conclusions:**

Aminoguanidine treatment counteracts the inflammation-induced nitrergic dysfunction and prevents dysmotility, both of which are independent of hyperglycaemia in BB rats. Nitrergic dysfunction may contribute to the pathophysiology of “low-grade inflammatory” FGIDs. Normoglycaemic BBDP rats may be considered a suitable animal model to study the pathogenesis of FGIDs.

## Introduction

Functional gastrointestinal disorders (FGID) are characterized by the presence of symptoms in the absence of organic, structural or metabolic underlying abnormalities that readily explain the symptoms [1]. The most prevalent FGID are functional dyspepsia and the irritable bowel syndrome. The pathogenesis of these disorders is poorly understood. An increasing number of publications points towards the presence of low-grade inflammatory changes in the mucosa and the enteric nervous system (ENS) of patients with FGID [2], [3]. These may be triggered by acute gastrointestinal infections, and hence they are often referred to as “post-infectious” FGIDs. However, many observations show ongoing low-grade inflammatory changes, which may lead to damage to the ENS, which coordinates gastrointestinal motility [4]–[9].

The relationship between mucosal inflammation, myenteric neuronal dysfunction and altered motor activity is poorly understood, and progress is hampered by the lack of suitable spontaneous animal models of inflammation-induced motor dysfunction. Observations in chemically induced intestinal inflammation by dextran sodium sulfate (DSS) and trinitrobenzene sulphonic acid (TNBS) have shown evidence of post-inflammatory dysfunction of nitrergic nerves [5], [10]–[14]. In keeping with the hypothesis that intestinal inflammation may preferentially affect nitrergic nerve function, we previously reported a high prevalence of impaired gastric accommodation attributable to a dysfunction at the level of gastric nitrergic neurons in patients with presumed post-infectious functional dyspepsia [4]. These observations suggest close interactions between acute inflammation, long-lasting nitrergic dysfunction and dysmotility in patients with “low-grade inflammatory” FGID.

The BioBreeding rat (BB rat) is a well-accepted spontaneous animal model for type 1 diabetes. All BB rats originate from a colony of outbred Wistar rats at the BioBreeding Laboratories in which spontaneous hyperglycaemia and ketosis appeared 30 years earlier [15], [16]. BB rats consist of diabetes resistant (BBDR) and diabetes prone (BBDP) strains. In the BBDP strain, 40–60% of animals develop diabetes between 60–120 days of age [17]. while the remaining BBDP and all the BBDR rats remain normoglycaemic for life. In diabetic BBDP rats, intestinal inflammation has been reported [18]. We previously reported that this could be linked to impaired nitrergic motor control since a decrease in neuronal nitric oxide synthase (nNOS) expression was found while purinergic neurotransmission, numbers of cholinergic neurons and the total number of neurons were not altered [19], [20]. These findings are compatible with what is found in other diabetic models, but recent preliminary observations from our group suggest that intestinal inflammation and nitrergic dysfunction are also present in normoglycaemic BBDP rats [21]. If confirmed, the normoglycaemic BBDP rat might constitute a spontaneous animal model for low-grade inflammatory intestinal dysmotility.

The susceptibility of nitrergic neurons to inflammation-induced damage has been attributed to expression of inducible nitric oxide synthase (iNOS) and oxidative stress [22], [23]. Nitrergic dysfunction is also observed in animal models of diabetes and has been attributed to oxidative stress and advanced glycosylation endproducts (AGEs) [24], [25]. Aminoguanidine (AG), an inhibitor of iNOS and AGE, has been shown to prevent loss of duodenal nNOS expression in streptozotocin induced diabetic rats [26].

The aim of the present study was 1) to study the prevalence of inflammation, altered nNOS expression and dysmotility in normoglycaemic and diabetic BBDP rats, and 2) to study the impact of inhibition of iNOS on these parameters in the normoglycaemic BBDP rats.

## Materials and Methods

### Animals

Breeding pairs of BBDP and BBDR were obtained from the Animal Resources Division of Health Canada, Ottawa, ON, Canada and further bred in the conventional animal facility of the University of Leuven, Belgium. In the remainder of the manuscript, BBDR rats will be referred to as “control rats”, normoglycaemic BBDP rats will be referred to as “inflamed rats”, hyperglycaemic BBDP rats will be referred to as “diabetic rats” (Table 1). Rats were housed in wire-meshed cages and had *ad libitum* access to drinking water and standard rat chow. Glycaemia was determined on tail blood at least every 3 days with a glucocard-memory-2 (Arkray, Kyoto, Japan). Daily amount of drinking was also monitored. Onset of diabetes was confirmed by monitoring the occurrence of hyperglycaemia (glycemia>250 mg/dL). Every 8 weeks, diabetic rats received subcutaneous insulin implants (Linplant, LinShin Canada Inc., Canada) to maintain hyperglycaemia and to prevent ketoacidosis (glycaemia between 300 and 400 mg dL^−1^).

**Table 1:** The BB rat population consists of a diabetes resistant strain (BBDR: control rats) and a diabetes prone (BBDP) strain.

Animal Group	Mutation of *Iddm1* and *Iddm2* gene[40]	Glycemia (mg/dl)	Amount of drinking (ml/day)
Bio Breeding Resistant (Control)	−	87.2±2.6	29.0±1.6
Normoglycemic Bio Breeding Prone rat (Inflamed rats)	+	98.6±3.4	35.4±1.6
Diabetic Bio Breeding Prone rat (Diabetic Rats)	+	411.9±9.0***	142.5±17.8***

However, although both normoglycaemic BBDP (inflamed rats) and diabetic BBDP (diabetic rats) carry the same genetic background, only diabetic rats demonstrated high glycaemia and polydipsia.

*** p<0.001, compared with both control rats and inflamed rats.

### Experimental design

Male or female diabetic rats and litter mate inflamed rats were sacrificed 16 weeks (inflamed rats: *n* = 7,diabetic rats: *n* = 7) after the onset of diabetes and were compared to age- and sex-matched control rats (*n* = 7). In our colony of BBDP rats, the prevalence of diabetes was 45–55%, which was in line with a previous report.[17] To study the impact of the relatively/fairly specific iNOS inhibitorAG, inflamed rats were given freshly prepared drinking water supplemented with AG (Sigma Aldrich, MO, U.S.A.) (*n* = 7) for 20 weeks. In a previous report, 1 g/L AG in the drinking water was used in a model of streptozocin-induced diabetes [27]. In our preliminary experiments, diabetic rats drank 5 times more water compared to control rats and inflamed rats. To compensate for this polydypsia, we added 5 g/L AG to the drinking water of inflamed rats.

Animals were euthanized by a sharp blow to the head and exsanguinated from the carotid arteries. The jejunum was harvested and divided into segments for the different assessments and kept in oxygenated (5% CO_2_/95% O_2_) ice cold Krebs solution (120.9 mmol L^−1^NaCl, 5.9 mmol L^−1^KCl, 1.2 mmol L^−1^MgCl_2_, 1.2 mmol L^−1^NaH_2_PO_4_, 14.4 mmol L^−1^NaHCO_3_, 11.5 mmol L^−1^Glucose, 2.5 mmol L^−1^CaCl_2_). A segment of jejunum was cut along the mesenteric axis and pinned flat in a sylgard-coated Petri-dish filled with ice-cold Krebs solution.

All procedures were approved by the Ethical Committee for Animal Experiments of the University of Leuven.

### Jejunal inflammation

Tissue myeloperoxidase (MPO) activity was measured with a previously described o-dianisidine method [28]. A segment of jejunum was processed with 0.9% NaCl.

After removal of mucosa, tissues were homogenized by Precellys 24 (Bertin technologies, Montigny-le-Bretonneux, France) in Hexadecyl Trimethyl Ammonium Bromide (HTAB) buffer, the homogenate was centrifuged (12500 g, 4°C; 30 min) and MPO activity of the supernatants was measured spectrophotometrically (Genesys 6: Thermo Fisher Scientific Inc., MA, U.S.A.) after addition of H_2_O_2_ and o-dianisidine hydrochloride.

For histological evaluation, a 1–2 cm long piece of jejunum was fixed in 4% formaldehyde for at least 24 h, and embedded in paraffin. Hematoxylin and Eosin (H&E)-staining was performed on 5 µm sections. Numbers of polymorphonuclear (PMN) cells per 50 ganglia and sum of thickness of circular muscle layer (CM) and longitudinal muscle layer (LM) were assessed light optically with calibrated grids by an experienced pathologist in a blinded manner.

### Quantitative Real-time RT-PCR

After removal of mucosa, tissues were homogenized by Precellys 24 in TRIzol (Invitrogen, CA, U.S.A.). Total RNA was extracted using the TRIzol extraction method. RNA solutions were purified by High pure RNA Isolation Kit (Roche Diagnostics, Mannheim, Germany) according to the manufacturer's instructions and reverse transcribed to complementary DNA (cDNA) with Transcriptor First Strand cDNA synthesis Kit (Roche Diagnostics). The quantitative real-time PCR reaction was run on a LightCycler 480 system (Roche Diagnostics) with LightCycler 480 SYBR Green I Master mix (Roche Diagnostics).

Specific primers for rat iNOS and hypoxanthine phosphoribosyltransferase 1 (HPRT1) were designed according to previous reports [29], [30]. One of the variants of nNOS, nNOSα can bind to the postsynaptic membrane via the postsynaptic density protein PSD-95 by its N-terminal PDZ domain, while another variant, nNOSβ, that lacks a PDZ domain, does not bind to PSD-95. Specific primers for nNOSα and nNOSβ were designed by targeting the PDZ domain of nNOSα cDNA (Gene bank: NM_052799) and the reported sequence of rat nNOSβ [31]. The following primers (forward and reverse) were used: for rat HPRT1, GCGAAAGTGGAAAAGCCAAGT and GCCACATCAACAGGACTCTTGTAG; for rat iNOS, ACCCAAGGTCTACGTTCAAGACA and CACATCCCGAGCCATGC; for rat nNOSα, GAGAACACGTTTGGGGTTCA and CTCCCACTTTGCGTTTGAAG; for rat nNOSβ, GGCAGCAGAGACCTCGAT and AGGACCACAGGAACGTTGTC. The real-time RT-PCR was performed with the following profile: 95°C for 10 min followed by 45 cycles of amplification (95°C for 10 s, 60°C for 15 s, 72°C for 10 s) followed by a melting curve program. An inter-run calibrator was used, and a standard curve was created for each gene to obtain PCR efficiencies. Relative target mRNA expression levels of all samples were expressed as a ratio to housekeeping, HPRT1 mRNA expression level. HPRT was selected as the housekeeping gene based on previous reports in BB-rats [32] and reports of a higher expression stability of HPRT among commonly used housekeeping genes [33].

### Immunohistochemistry

Whole mounts were prepared after removal of mucosa and submucosa by dissection using watchmaker's forceps and scissors to obtain preparations of circular and longitudinal smooth muscle layer and adherent myenteric plexus. Whole mount preparations were fixed in 4% paraformaldehyde for 40 minutes at room temperature (RT). After 4 times washing with PBS (1.5 mmol L^−1^ KH_2_PO_4_, 2.7 mmol L^−1^ Na_2_HPO_4_.2H_2_O and 155.2 mmol L^−1^NaCl; pH 7.2), the tissues were stored in PBS containing 0.03% sodium azide until usage.

The whole mount preparations were processed for permeabilization and blocking of non-specific binding sites for 2 hr at RT with 0.5% triton-X100 and 4% donkey and goat serum in 0.1 mol L^−1^ PBS. The tissues were incubated (24 h; 4°C) with the primary antibody of rabbit anti-nNOS C-terminal (1∶400; Santa Cruz Biotechnology, Santa Cruz, CA, USA), mouse anti-Hu-C/D (1∶500; Molecular Probes, Eugene, OR, USA) and chicken anti-neurofilament 200(1∶5000;Abcam, Cambridge, UK). After washing with PBS, secondary antisera were applied: goat anti-chicken Alexa594 (1∶1000; Invitrogen, Life Technologies Corp., Carlsbad, CA, USA), donkey anti-mouse Alexa488 (1∶1000; Invitrogen) and donkey anti-rabbit AMCA (1∶250; Jackson Immuno Research Labs, West Grove, PA, USA).

The stained whole mount preparations were visualized using a fluorescence microscope (BX41; Olympus, Tokyo, Japan) equipped with a digital camera (XM-10; Olympus).

### Organ bath

Integrated intestinal motility was evaluated in an organ bath with video-imaging analysis as reported before [34]. Briefly, 5 cm segments of rat jejunum were suspended in pairs in a double chamber organ bath filled with oxygenated Krebs solution at 37°C. Oral and aboral intraluminal pressure could be regulated by adjusting the height of Krebs-filled syringes in series connected to the intestinal segments. Intestines were allowed to equilibrate for 30 min and imaged for 90 s (10 Hz) at baseline (0 cm H_2_O) and after increased oral pressure (3 cm H_2_O) for 5 minutes. Two segments were imaged simultaneously. Due to the background of the organ baths, the intestine could be detected automatically by threshold operations in each individual image of the sequence. In contrast to similar measurements in guinea pigs [34], no peristaltic activity was observed in rats, so we monitored the diameter and length of the intestine along its 5 cm length using custom written software routines in Igor Pro (WaveMetrics, Inc., Portland, OR, USA).

### Toluidine blue staining

Tissues were fixed in 4% (w/v) paraformaldehyde and embedded in paraffin.

Transverse sections (5 µm) were stained for mast cells by 0.1% of toluidine blue (Sigma-Aldrich) for 1 hour and washed in PBS.

### Data presentation and statistical analysis

Data are expressed as mean and SEM, normally. However, if the distributions were not normal, data are expressed as median [first–third quartile]. Distributions were tested by F-test or Bartlett test. If the distributions were normal, parametric tests (Student's *t*-test or One-way ANOVA followed by Dunnett *post hoc* test) were applied. Otherwise, non-parametric tests (Mann-Whitney U test or Kruskal-Wallis test followed by Steel *post hoc* test) were applied for analysis. Statistical analysis was performed using Excel (Microsoft, Redmond, WA, USA). Significance was accepted at the *p*<0.05 level.

## Results

### Intestinal inflammation

**Figure 1:**
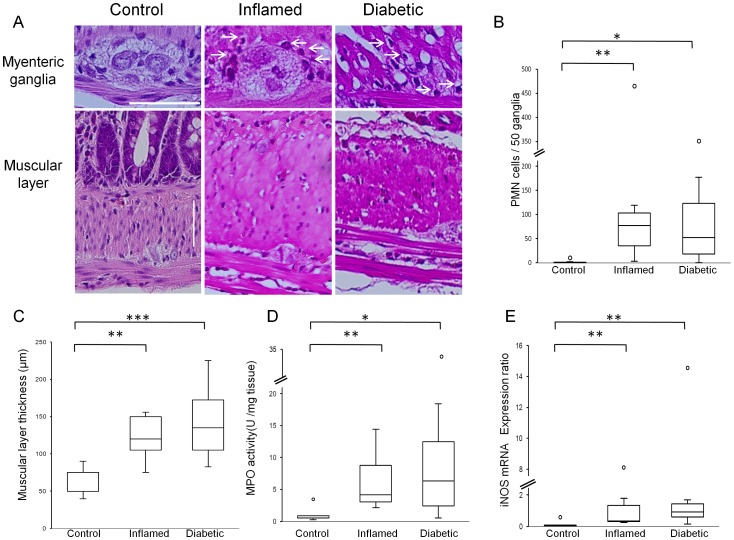
Intestinal inflammation in BB rats. Representative images of the myenteric ganglia and the muscular layer of the jejunum as stained by H&E (A). Intra-ganglionic infiltration of PMN cells (arrows) and thicker muscular layers were observed in both inflamed and diabetic rats. All scale bars: 50 µm. Numbers of PMN cells per 50 myenteric ganglia (B) in both inflamed and diabetic rats increased, compared to control rats (**p<0.01, *p<0.05, respectively). Sum of circular and longitudinal muscular layer thickness (C) in both inflamed and diabetic rats increased, compared to control rats (**p<0.01, ***p<0.001, respectively by One-way ANOVA followed by Dunnett *post hoc* test). Jejunal MPO activity (D) in both inflamed rats and diabetic rats increased, compared to control rats (**p<0.01, *p<0.05,). Jejunal iNOS mRNA expression (E) in both inflamed and diabetic rats increased, compared to control rats (**p<0.01). If not mentioned, *p* values were obtained by Kruskal-Wallis test followed by Steel *post hoc* test.

To test whether our preliminary findings, which indicated that intestinal inflammation is independent of overt diabetes, could be confirmed, we examined various inflammatory parameters. First, using H&E stainings, intra-ganglionic infiltration of PMN cells (Fig. 1 B) in both inflamed rats and diabetic rats was observed (Fig. 1 A). Because of the difficulties to distinguish neutrophils from eosinophils in rats, based on H&E-staining only, the total number of PMN cells was quantified. However, the increased jejunal neutrophil specific MPO activity in both inflamed and diabetic rats (Fig. 1 D), suggests an infiltration of neutrophils into the myenteric ganglia of inflamed and diabetic rats. Finally, we also observed increased iNOS mRNA expression in the neuromuscular layer of both inflamed and diabetic rats (Fig. 1 E). These changes indicate myenteric ganglionitis in both groups of rats, independent of the presence of hyperglycaemia. Moreover, similar to previous reports on post-inflammatory intestinal changes in rodents[5], [31], an increased smooth muscle layer thickness (Fig. 1 A, C) was observed in inflamed as well as in diabetic rats.

### Changes in myenteric neurons

**Figure 2:**
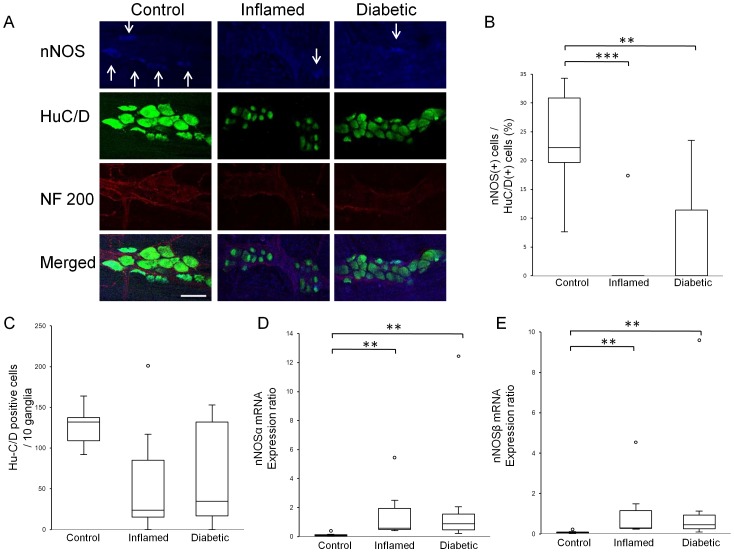
Changes of myenteric neurons in BB rats. Representative images of immunohistochemical stainings of myenteric ganglia by anti-nNOS antibody (blue), anti-Hu-C/D antibody (green) and anti-neurofilament 200 antibody (red) in BB rats (A). Arrows indicate nNOS positive neuronal cells. Scale bar: 50 µm. Hu-C/D positive cells (C) in both inflamed rats and diabetic rats did not change significantly. However, the ratio of nNOS positive cells to Hu-C/D positive cells (B) in both inflamed and diabetic rats decreased, compared to control rats (***p<0.001, ***p<0.001, respectively by One-way ANOVA followed by Dunnett *post hoc* test). Both nNOSα (D) and nNOSβ (E) mRNA expression in inflamed rats and diabetic rats increased, compared to control rats (**p<0.01). If not mentioned, *p* values were obtained by Kruskal-Wallis test followed by Steel *post hoc* test.

To address whether changes in nitrergic dysfunction are also independent of the presence of hyperglycaemia in diabetes-prone BB rats, we compared the number of nitrergic neurons between the 3 experimental groups. Although the number of Hu-C/D positive cells in the myenteric plexus was not altered (Fig. 2A,C), we found a decreased ratio of nNOS positive cells to Hu-C/D positive cells (Fig. 2 B) in both inflamed and diabetic rats. Interestingly, the mRNA expression levels of nNOSα (Fig. 2 D) and nNOSβ (Fig. 2 E) increased in both diabetes-prone groups.

### Intestinal dysmotility

**Figure 3:**
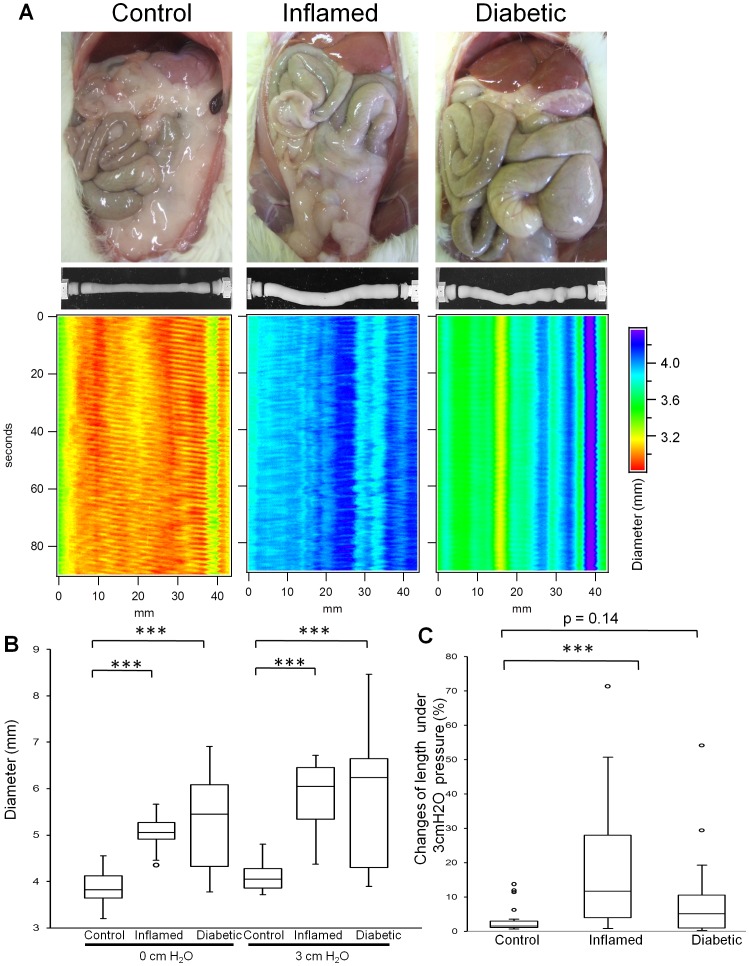
Dysmotility in BB rats. Macroscopic abdominal findings in BB rats (A). Dilated intestines were observed in both inflamed rats and diabetic rats. Representative images of organ bath video analysis and spatiotemporal maps depicting the diameter of the intestinal segment upon an oral 3 cm H_2_O stimulus (A). The short-lived aborally propagating contractions that are clearly observed in control gut are impaired in both inflamed and diabetic animals. Dilated and elongated intestines were observed in both inflamed and diabetic rats. The jejunal diameter (B) in oral 0 and 3 cm H_2_O stimulus of both inflamed and diabetic rats were increased compared to control rats (***p<0.001). Changes in jejunal length (C) under 3 cm H_2_O stimulus in inflamed rats were increased compared to control rats (***p<0.001). In diabetic rats, this increase was not statistically significant (*p* = 0.14). Both *p* values were obtained by Kruskal-Wallis test followed by Steel *post hoc* test

When examined macroscopically, dilated intestines were observed in both inflamed and diabetic rats (Fig. 3 A). These signs of intestinal dysmotility were confirmed *in vitro*. Spatiotemporal maps show prominent short-lived aborally propagating contractions (myogenic ‘ripples’ [35]) observed in jejunal segments of control rats. The intestine of inflamed animals was dilated as seen from the blue color and ripples appeared disorganized and were almost absent in diabetic animals. Furthermore, both an oral 0 and 3 cm H_2_O stimulus induced abnormal dilation in both inflamed and diabetic rats (Fig. 3 A, B), and elongation in intestinal segments obtained from inflamed rats (Fig. 3 A, C). Taken together, these findings indicate impaired intestinal CM and LM function in both groups.

### Characterization of inflammatory cell infiltrate

**Figure 4:**
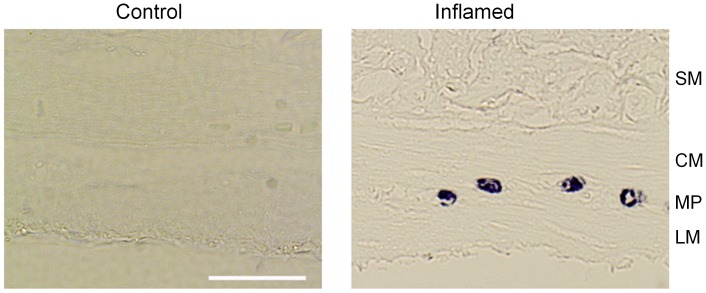
Mast cell infiltration in myenteric plexus of inflamed rats. In the myenteric plexus of control rats, no toluidine blue positive cells were found (left). In the myenteric plexus of inflamed rats, toluidine blue positive mast cells were found (right). Scale bar: 100 µm. SM: Sub-mucosal layer, CM: Circular muscle layer, MP: Myenteric plexus LM: Longitudinal muscle layer.

Toluidine blue staining confirmed the presence of mast cell infiltration in the myenteric plexus layer of inflamed rats (Figure 4 A, B).

### Effect of AG treatment

**Figure 5:**
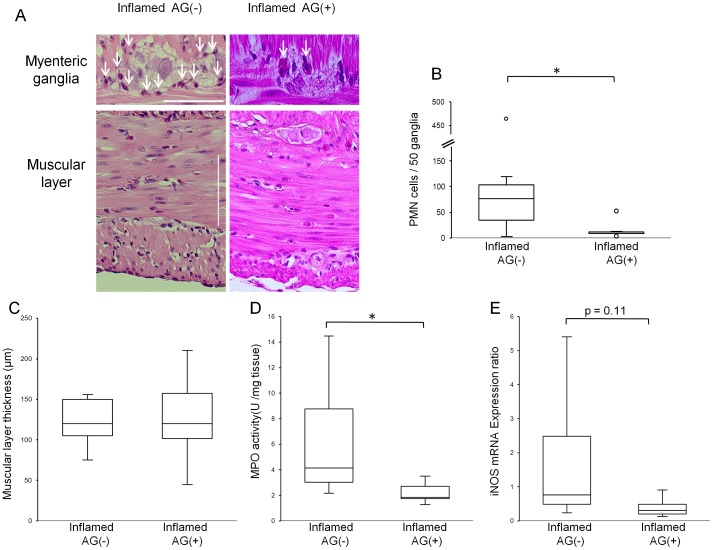
Effect of AG on intestinal inflammation in inflamed rats. Representative images of the myenteric ganglia and the muscular layer by H&E stainings in inflamed rats with or without AG treatment (A). All scale bars: 50 µm. AG treatment prevented infiltration of PMN cells in myenteric ganglia (arrows). AG treatment decreased numbers of PMN cells per 50 myenteric ganglia (B) (*p<0.05). However, AG treatment had no effect on the muscular layer thickness (C) (by Student's *t*-test). AG treatment dercreased jejunal MPO activity (D) (*p<0.05) and jejunal iNOS mRNA expression (E), however, not statistically significant (*p* = 0.11). If not mentioned, *p* values were obtained by Mann-Whitney U test.

**Figure 6:**
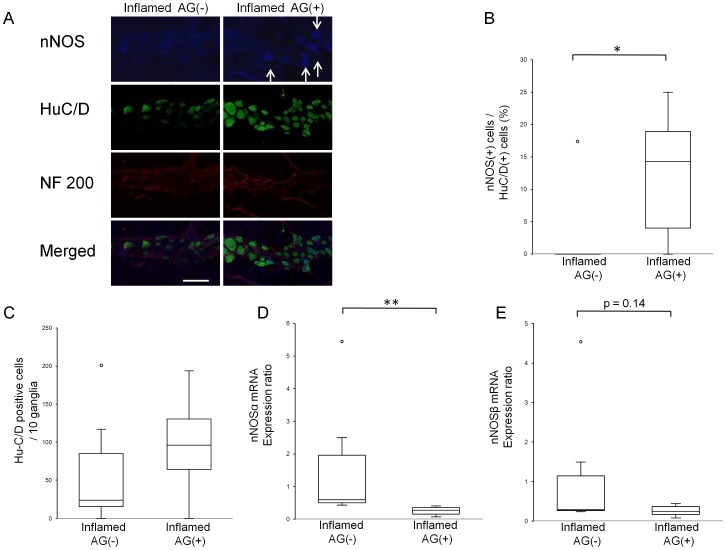
Effect of AG on myenteric neurons in inflamed rats. Representative images of immunohistochemical stainings of myenteric ganglia by anti-nNOS antibody (blue), anti-Hu-C/D antibody (green) and anti-neurofilament 200 antibody (red) in inflamed rats with or without AG treatment (A). Arrows indicate nNOS positive neuronal cells. Scale bar: 50 µm. AG had no effect on Hu-C/D positive cells (C) (*p* = 0.35 by Student's *t*-test). However, AG treatment increased the ratio of nNOS positive cells to Hu-C/D positive cells (B). (*p<0.05 by Student's *t*-test). Enhanced nNOSα mRNA expression (D) (*p<0.05) and enhanced nNOSβ mRNA expression (E) are decreased by AG treatment, however, not statistically significant (*p* = 0.14). If not mentioned, *p* values were obtained by Mann-Whitney U test.

**Figure 7:**
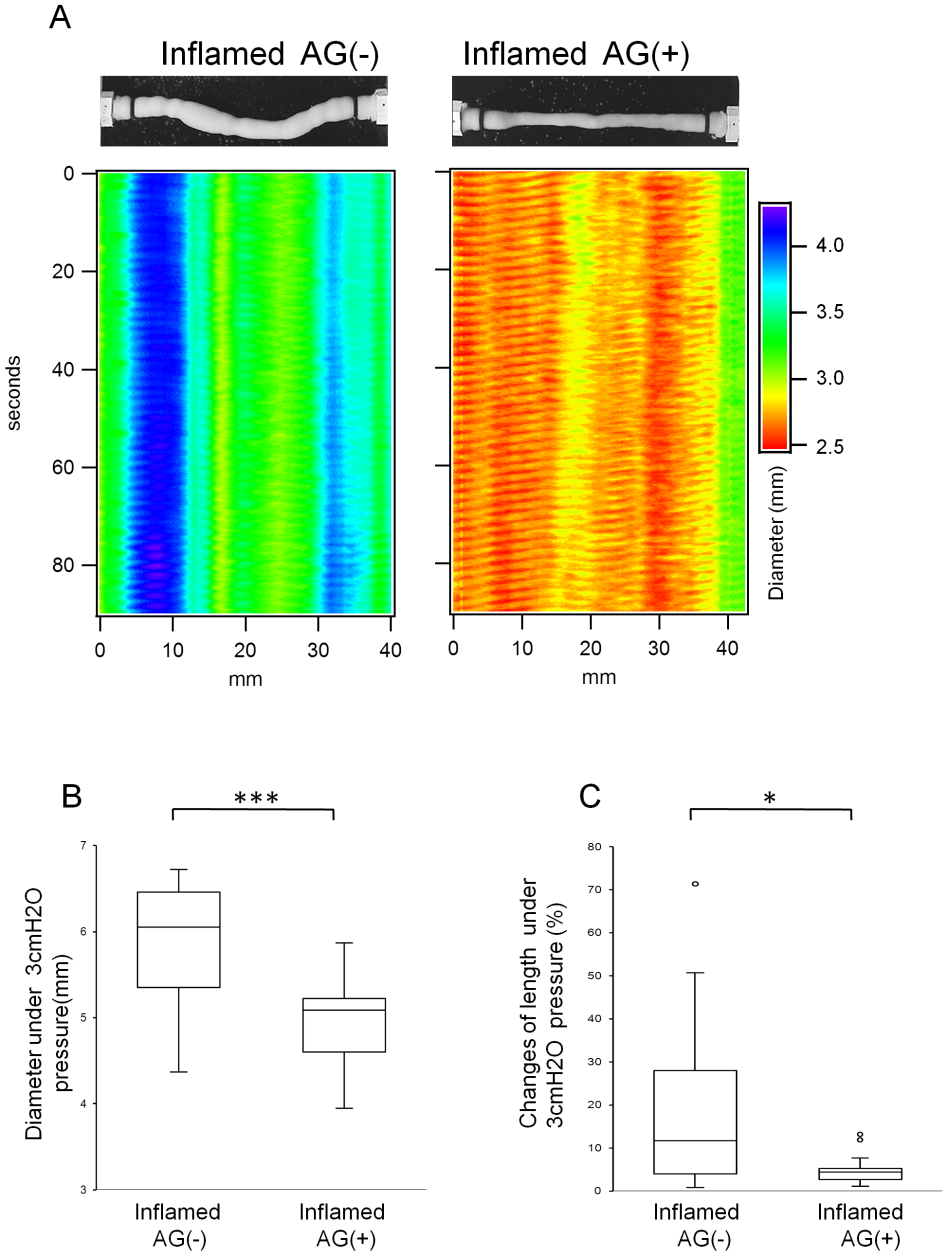
Effect of AG on dysmotility in inflamed rats. Representative images of organ bath video analysis and spatiotemporal maps depicting the diameter of the intestinal segment under oral 3_2_O stimulus in inflamed rats with or without AG treatment (A). AG treatment partially reinstalled the short-lived propagating contractions that were prominent in control. AG treatment reduced jejunal diameters (B) in inflamed rats (***p<0.001 by Student's *t*-test) and also abolished the changes in jejunal length (C) in inflamed rats (*p<0.05 by Mann-Whitney U test).

AG treatment reduced the number of infiltrated PMN cells in myenteric ganglia (Fig. 5 A,B) and jejunal neuromuscular MPO activity (Fig. 5 D) in inflamed rats. Jejunal iNOS mRNA expression (Fig. 5 E) decreased after AG treatment, however without reaching statistical significance. The muscular layer thickness (Fig. 5 A, C) was not changed by AG treatment, nor did it affect the number of Hu-C/D positive cells (Fig. 6 A, C). However, AG treatment partially restored the proportion of nitrergic neurons (Fig. 6 A, B). In addition, AG treatment attenuated enhanced jejunal nNOSα mRNA expression (Fig. 6 D) and lowered the enhanced jejunal nNOSβ mRNA expression – albeit not statistically significant- (Fig. 6 E). Moreover, AG treatment partially reinstalled the short-lived aborally propagating contractions and limited the dilation (Fig. 7 A and B) and also prevented elongation of jejunal segments *in vitro* (Fig. 7 A and C) observed in response to an oral 3 cm H_2_O pressure stimulus. This indicates that AG treatment improved circumferential and longitudinal dysmotility in inflamed rats.

### Longitudinal smooth muscle strip experiments

Incubation of muscle strips from control animals with AG 10^−6^ and 10^−4^ M did not affect the EFS induced relaxation (ANOVA, *p* = 0.33) Subsequent addition of L-NAME significantly decreased muscle relaxation (ANOVA P<0.0001) at all frequencies between 2 and 16 Hz (Figure S1.A).

No differences in muscle relaxation were observed between AG treated animals and age-matched controls (Figure S1.B). In addition, incubation of muscle strips with L-NAME strongly decreased muscle relaxation (ANOVA, p = 0.0005) at all frequencies between 2 and 8 Hz. No differences ACh-induced maximal contraction or relaxation to NG were observed between treated and non-treated animals (*p* = 0.50).

## Discussion

In the present study, we demonstrated that BBDP rats present with intestinal inflammation and nitrergic dysfunction, independent of the presence of diabetes. Some of these changes were already reported in diabetic BBDP rats, but the issue whether or not they were influenced by hyperglycaemia is unknown yet.[19] We report for the first time that normoglycaemic BBDP rats also show evidence of inflammatory enteric neuropathy, thereby establishing that this is not merely a complication of diabetes. Consequently, normoglycaemic BBDP can be considered the first spontaneous animal model which allows studying the mechanism underlying inflammation-induced intestinal dysmotility or so-called “low-grade inflammatory” FGIDs.

Törnblom H et al. investigated full-thickness biopsy specimens from the proximal jejunum in patients with intestinal dysmotility (referred to as “severe IBS”) [6]. They found presence of low-grade inflammation, characterized by infiltration of lymphocytes, and neuronal degeneration in the myenteric plexus in the jejunum. We and others also reported low-grade inflammation in mucosal biopsies of patients with functional dyspepsia [7]–[9]. These data suggest a role for inflammatory changes in the small intestine (jejunum and duodenum) contributing to the pathogenesis of severe FGIDs. These observations led us to explore the potential of the jejunum from the non-diabetic BB rat as a model to study the role of low-grade inflammation in intestinal dysmotility.

Recently, we reported impaired gastric accommodation in this model. Several studies in animals and patient groups have provided evidence for impaired nitrergic motor control in patients with and animal models of FGID [4] [19] [20]. We recently reported loss of nitrergic neurons contributing to an impaired gastric accommodation in the BB-rat model [36], which prompted us to focus on the nitrergic neuron population in this model.

We found decreased nNOS mRNA expression and decreased nitrergic nerves by immunohistochemistry. In a previous study, we showed that this was associated with decreased nNOS protein expression as assessed by Western blot [19]. The myenteric ganglionitis in BBDP rats appears to be neutrophil dominant, and this differs from findings in patients with severe IBS, patients with chronic intestinal pseudo-obstruction and diabetic gastroparesis, where lymphocytes were reported as the dominant cell type [6], [37], [38]. However, due to polygenic changes, BBDP rats display a number of alterations in leukocyte populations [39]. Lymphopenia in BBDP rats is likely to be the reason for neutrophil dominance in this model of myenteric ganglionitis. In our model, there is absence of lymphocytes in the myenteric plexus (CD4 and CD8 stainings are negative:data not shown) which makes sense taking into account the lymphopenic nature of the BB-rat. Increased MPO and granulocyte infiltration are characteristic and also observed in tissues of patients with relapsing inflammatory bowel diseases which are defined as chronic inflammatory diseases. Moreover, increased neutrophil involvement in patients with IBS was reported [40]. Chronic inflammation can be considered as an important pathophysiological factor of FGID.

Previous studies have already shown improvement of nitrergic dysfunction by AG in the streptozotocin model of diabetes, which was attributed to the ability of AG to inhibit hyperglycemia- associated AGEs [27]. In the present study, we demonstrated that AG treatment is able to counteract the inflammation-induced nitrergic dysfunction and to prevent intestinal dysmotility in the normoglycaemic inflamed rats. Besides its effect on AGEs, AG is also known to be an inhibitor of iNOS which could contribute to its effect on nitrergic dysfunction [22], [23]. AG is reported to be 10 times more selective for iNOS than for nNOS [36], [41]. Its inhibiting effect depends of course on the final concentration in the targeted tissue. To investigate selective or non-selective effects of AG on gastrointestinal motility we performed muscle strip experiments but did not observe acute contractility changes (Methods S1). In these studies we showed that AG had no direct effect on EFS-induced relaxation. This indicates that improvement of inflammation by AG through iNOS inhibition, and through another mechanism, induced the improvement in dysmotility.

Studies in enteric neuron cultures suggest that iNOS overexpression down-regulates nNOS expression through oxidative stress [19]. In line with such a mode of action, AG decreased nitrergic dysfunction with mild attenuation of enhanced iNOS mRNA expression in inflamed rats. In addition, AG has an *in vitro* antioxidant effect [42]. Thus, besides inhibition of iNOS, the antioxidant effect of AG may also have contributed to preservation of nitrergic motor control in normoglycaemic inflamed rats in the present study.

Unlike its effect on nNOS expression and motor control, AG treatment did not restore the increased muscular layer thickness in inflamed rats. This may be due to the time point of starting AG treatment, which was only initiated after confirmation of the non-development of diabetes. It is conceivable that irreversible changes in muscular layer thickness may have occurred before this time point. A detailed time course study would be needed to elucidate the time point of development of increased muscular layer thickness in this model.

Because NO is well known to be an inhibitory neurotransmitter in the ENS, improvement of dysmotility by AG treatment may seem paradoxical at first. NO enhances not only inhibitory synaptic transmission but also neuronal excitability in guinea-pig submucous plexus [43]. Moreover, changes in nitrergic nerve function are confounded by the presence of different isoforms and splice variants of nNOS [44]. In the rat small intestine, among splice variants of nNOS, only nNOSα and nNOSβ are reported as functionally active variants of nNOS [45]. Compared with nNOSα, nNOSβ has 80% catalytic activity for NO production [44]. In nerve varicosities of the rat CM, both membrane-bound nNOSα and cytosolic nNOSβ are expressed, while in the LM only cytosolic nNOSβ is expressed in nerves [45]. In isolated LM strips, NO reportedly caused only contraction [46]. Due to limitation of our anti-nNOS C-terminal antibody, evaluation of protein level expression of each nNOS variant is difficult. The increase in mRNA expression of nNOS variants could represent a compensatory effect for the decrease in the proportion of nitrergic neurons. However, accompanied with attenuation of enhanced mRNA expression of each nNOS variant, the total proportion of intra-ganglionic nNOS positive cells in inflamed rats was restored by AG treatment. Thus, enhancement of protein level expression of nNOSβ can be presumed, which may have contributed to improvement of longitudinal dysmotility. Of course, adequate motor control depends on a coordination of inhibitory and excitatory motor pathways [47], and restoring this balance by preserving nitrergic function is another pathway that may contribute to the AG-induced improvement in motor control in our model.

In conclusion, we confirmed an interaction between inflammation, nitrergic dysfunction and intestinal dysmotility in BBDP rats, regardless of the presence of hyperglycaemia. Dysmotility related to inflammatory nitrergic dysfunction improved after AG treatment. These data support the hypothesis that inflammation-induced nitrergic dysfunction may contribute to the pathophysiology of presumed “low-grade inflammatory” FGIDs, and propose the normoglycaemic BBDP as a suitable spontaneous animal model to study such condition.

## Supporting Information

Figure S1 **Effect of aminoguanidine on EFS-induced muscle relaxation.** EFS induced muscle relaxation was measured in the organ bath. (A) Relaxatory response at different frequencies (1–32 Hz) was measured under NANC (black dots), NANC + Aminoguanidine 10^−6^ M (AG, white squares), NANC + AG 10^−4^ M (black triangles) and NANC + AG 10^−4^+ L-NAME 10^−5^ M (white dots). (*p<0.05, *** *p*<0.001, **** *p*<0.0001). (B) Comparison of EFS induced muscle relaxation between AG treated (black dots; n = 18, N = 6) and non-AG treated (white dots; n = 15, N = 5) control rats. Relaxation amplitude is expressed as percentage of the total relaxation induced by nitroglycerine 10^−5^ M.
(TIF)

Methods S1 **Longitudinal smooth muscle strip experiments.**
(DOCX)
