# Supplementary figures and images for: A Spontaneous Animal Model of Intestinal Dysmotility Evoked by Inflammatory Nitrergic Dysfunction
Source: PLoS One. 2014 May 12;9(5):e95879. doi: 10.1371/journal.pone.0095879 (PMC4018386; doi:10.1371/journal.pone.0095879)

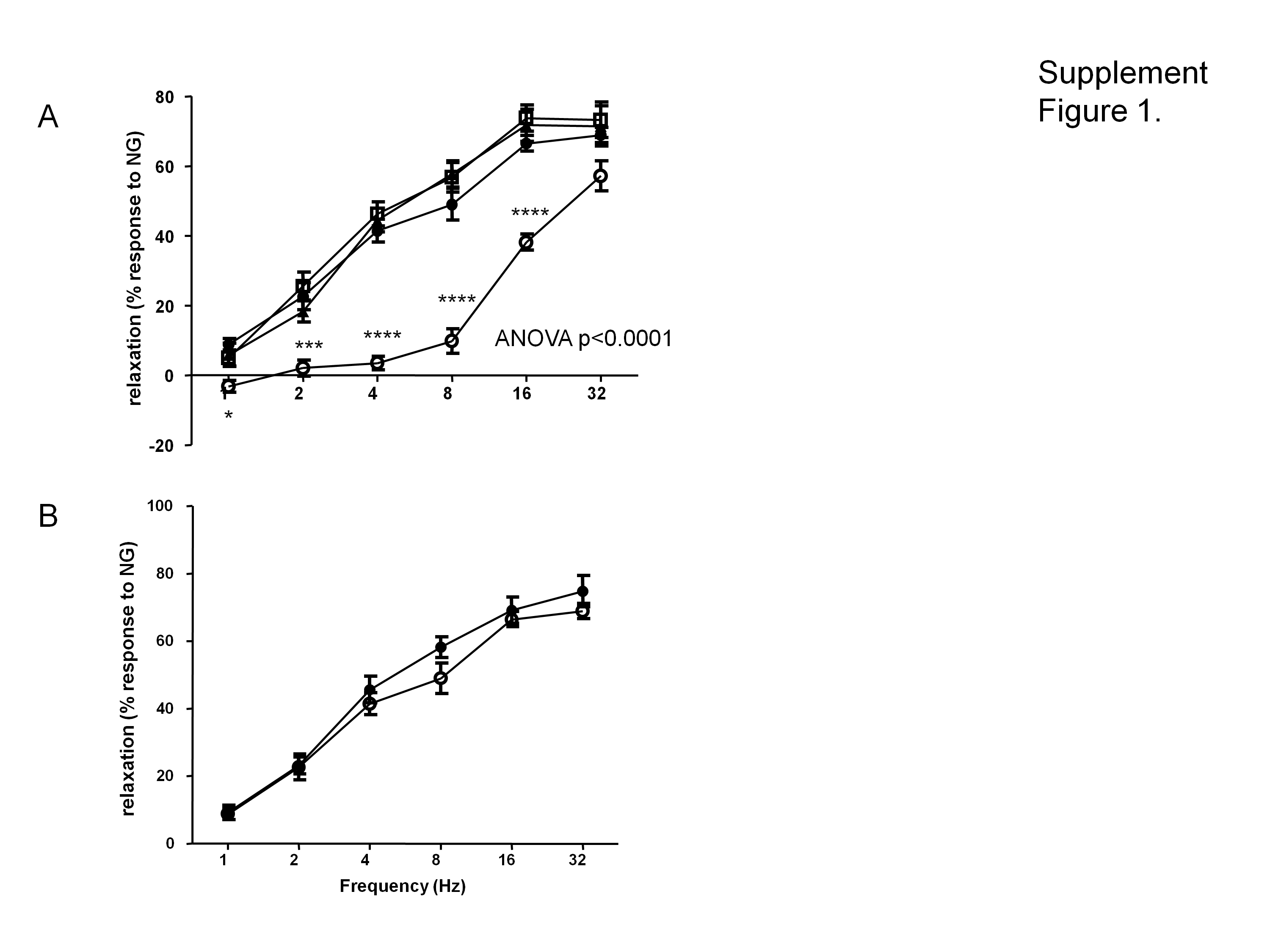

Supplement: Figure S1 — Effect of aminoguanidine on EFS-induced muscle relaxation. EFS induced muscle relaxation was measured in the organ bath. (A) Relaxatory response at different frequencies (1–32 Hz) was measured under NANC (black dots), NANC + Aminoguanidine 10−6 M (AG, white squares), NANC + AG 10−4 M (black triangles) and NANC + AG 10−4+ L-NAME 10−5 M (white dots). (*p<0.05, *** p<0.001, **** p<0.0001). (B) Comparison of EFS induced muscle relaxation between AG treated (black dots; n = 18, N = 6) and non-AG treated (white dots; n = 15, N = 5) control rats. Relaxation amplitude is expressed as percentage of the total relaxation induced by nitroglycerine 10−5 M. (TIF) [file pone.0095879.s001.tif]
