# Supplementary material for: A Spontaneous Animal Model of Intestinal Dysmotility Evoked by Inflammatory Nitrergic Dysfunction
Source: PLoS One. 2014 May 12;9(5):e95879. doi: 10.1371/journal.pone.0095879 (PMC4018386; doi:10.1371/journal.pone.0095879)
Supplement: Methods S1 — Longitudinal smooth muscle strip experiments. (DOCX) [file pone.0095879.s002.docx]

**Supplementary materials and methods**

**Methods**

**Longitudinal smooth muscle strip experiments**

Longitudinal muscle strip experiments were performed to evaluate the effect of short term (3 weeks) and acute AG administration on nitrergic motor neuron function in control animals. Muscle strip experiments were performed as previously described [19]. Briefly, mucosal and submucosal tissue was removed from the jejunum and muscle strips (length ±15 mm) were cut and suspended along their longitudinal axis in an organ bath filled with Krebs solution. After an equilibration period of 40 min at a resting tension of 1g, acetylcholine (ACh) 10^-4^ M was added to measure maximal contraction of the muscle strips. From then on, all experiments were performed under non-adrenergic non-cholinergic (NANC, atropine 10^-6^ M and guanethidine 4x10^-6^ M) conditions. Strips were pre-contracted with serotonin (5-HT) 10^-6^ M prior to electrical field stimulation (EFS, 1–32 Hz, pulse 0.35 ms, train 10 s, 8 V). EFS was applied by means of an electrical stimulator (Model S88; Grass Instruments Co, West Warwick, RI, USA) and a power booster (Stimu-Splitteer II; Med-Lab Instruments, Loveland, CO, USA). EFS-induced relaxation under NANC conditions was evaluated in muscle strips from non-treated and AG pre-treated (5g/L in the drinking water for 3 weeks) animals. To evaluate the direct effect of AG on smooth muscle relaxation, separate experiments were performed with sequential addition of AG 10^-6^ M and AG 10^-4^ M to the organ bath with muscle strips from untreated control animals. After 15 minutes incubation, muscle strip relaxation was induced via EFS in the presence and absence of the nitric oxide synthase inhibitor Nitro-L-arginine methyl ester (L-NAME) 3x 10^-4^ M to evaluate the nitrergic contribution. The amplitude of relaxation during the stimulation period (on-response) was measured and expressed as percentage of the maximal relaxation induced by nitro-glycerine (NG) 10^-5^ M. All results were corrected for the cross-sectional area of the individual muscle strips.
